# Immune-Associated Gene Signatures and Subtypes to Predict the Progression of Atherosclerotic Plaques Based on Machine Learning

**DOI:** 10.3389/fphar.2022.865624

**Published:** 2022-04-26

**Authors:** Yujia Yang, Xu Yi, Yue Cai, Yuan Zhang, Zhiqiang Xu

**Affiliations:** ^1^ Department of Neurology and Centre for Clinical Neuroscience, Daping Hospital, Army Medical University (Third Military Medical University), Chongqing, China; ^2^ Department of Cardiology, Xijing Hospital, Fourth Military Medical University, Xi’an, China

**Keywords:** atherosclerosis, atherosclerotic plaque, immune-associated genes, characteristic genes, immune subtype

## Abstract

**Objective:** Experimental and clinical evidence suggests that atherosclerosis is a chronic inflammatory disease. Our study was conducted for uncovering the roles of immune-associated genes during atherosclerotic plaque progression.

**Methods:** Gene expression profiling of GSE28829, GSE43292, GSE41571, and GSE120521 datasets was retrieved from the GEO database. Three machine learning algorithms, least absolute shrinkage, and selection operator (LASSO), random forest, and support vector machine–recursive feature elimination (SVM-RFE) were utilized for screening characteristic genes among atherosclerotic plaque progression- and immune-associated genes. ROC curves were generated for estimating the diagnostic efficacy. Immune cell infiltrations were estimated via ssGSEA, and immune checkpoints were quantified. CMap analysis was implemented to screen potential small-molecule compounds. Atherosclerotic plaque specimens were classified using a consensus clustering approach.

**Results:** Seven characteristic genes (TNFSF13B, CCL5, CCL19, ITGAL, CD14, GZMB, and BTK) were identified, which enabled the prediction of progression of atherosclerotic plaques. Higher immune cell infiltrations and immune checkpoint expressions were found in advanced-stage than in early-stage atherosclerotic plaques and were positively linked to characteristic genes. Patients could clinically benefit from the characteristic gene-based nomogram. Several small molecular compounds were predicted based on the characteristic genes. Two subtypes, namely, C1 immune subtype and C2 non-immune subtype, were classified across atherosclerotic plaques. The characteristic genes presented higher expression in C1 than in C2 subtypes.

**Conclusion:** Our findings provide several promising atherosclerotic plaque progression- and immune-associated genes as well as immune subtypes, which might enable to assist the design of more accurately tailored cardiovascular immunotherapy.

## Introduction

Atherosclerosis is a systematic, progressive, inflammatory disease, which remains a leading cause of mortality and morbidity globally (Lorenzo et al., 2021). Chronic accumulation of vascular occlusive plaques within the subendothelial intimate layer of large- and medium-sized arteries leads to severe stenosis, and thus limits blood flow as well as triggers severe hypoxia (Libby, 2021). Myocardial infarction and stroke are frequent complications resulting from spontaneous thrombotic vascular occlusion (Qiao et al., 2020). Atherosclerotic plaque formation is a slow process that provides a window of opportunity for presymptomatic diagnosis (Nayor et al., 2021). Invasive intravascular imaging enables to assess vessel stenosis and wall thickness completely and in detail, while non-invasive medical imaging is more conducive to non-invasively identify vulnerable plaques and more accurately stratify cardiovascular risk (Lenz et al., 2020). Hence, it is urgent to develop advanced molecular tools for risk stratification of atherosclerotic plaques.

Atherosclerotic lesions are composed of cells from innate and adaptive immune systems (Tong et al., 2021). The immune mechanism is a crucial driver of the progression of atherosclerotic plaques and ruptures, which has been a target to identify vulnerable plaques (Tong et al., 2021). It enables to orchestrate all stages within the life cycle of atherosclerotic plaques. The initiation of atherosclerosis involves endothelial activation, which recruits leukocytes to the arterial intima, in which they are linked to lipoprotein and its derivative, and thus accumulate in the layer (Mushenkova et al., 2020). The long-term and slow progression of atherosclerosis involves persistent immune response, with intermittent acute activation episodes resulting from extravascular damage or immune activation at the site of infection or subclinical destruction of plaques (Lenz et al., 2021). The single-cell immune landscape of the human atherosclerotic plaques has uncovered that innate and adaptive immune cells in plaques show associations with cerebrovascular events (Fernandez et al., 2019). In a previous bioinformatics analysis, immune cell infiltrations and immune-associated pathways participate in atherosclerotic plaque progression (Tan et al., 2021). These findings highlight the crucial role of immune mechanisms in atherosclerosis. Here, we applied three machine learning algorithms, least absolute shrinkage and selection operator (LASSO), random forest, and support vector machine–recursive feature elimination (SVM-RFE) to determine characteristic genes among atherosclerotic plaque progression- and immune-associated genes, which enabled the prediction of the progression of atherosclerotic plaques. Moreover, we proposed a novel classification of atherosclerotic plaques containing immune and non-immune subtypes.

## Materials and Methods

### Microarray Datasets and Data Preprocessing

Raw gene expression profiling of atherosclerosis patients was accessed from GSE28829 (Döring et al., 2012), GSE43292 (Ayari and Bricca, 2013), GSE41571 (Lee et al., 2013), and GSE120521 (Mahmoud et al., 2019) datasets of the Gene Expression Omnibus (GEO; https://www.ncbi.nlm.nih.gov/gds/). In the GSE28829 dataset, there are 13 early carotid atherosclerotic plaque specimens (pathological intimal thickening and intimal xanthoma) and 16 advanced carotid atherosclerotic plaque specimens (thin or thick fibrous cap atheroma), detected by the Affymetrix Human Genome U133 Plus 2.0 Array. The GSE43292 dataset includes 32 early-stage and 32 advanced-stage carotid atherosclerotic plaque specimens, detected by the Affymetrix Human Gene 1.0 ST Array. The GSE41571 dataset contains five ruptured atherosclerotic plaque specimens and six stable atherosclerotic plaque specimens, detected by the Affymetrix Human Genome U133 Plus 2.0 Array. The GSE120521 dataset comprises four stable and four unstable atherosclerotic plaque specimens. Due to the similarity of sequencing methods, stages of plaque investigated, and study design/comparison between the GSE28829 and GSE43292 datasets, the expression profiling of the aforementioned datasets was merged as the discovery set and batch effects were directly adjusted for batch effects utilizing Combat function of sva package (Leek et al., 2012). Principal component analysis (PCA) was applied for evaluating the performance of the Combat function. The GSE41571 and GSE120521 datasets were utilized as the external verification sets. The probe ID for each gene was transformed into a gene symbol. If a gene symbol corresponded to several probe IDs, the average expression value of the probe IDs was calculated as the representative expression value of the gene.

### Analysis of Atherosclerotic Plaque Progression- and Immune-Associated Genes

The list of 1,242 immune-associated genes was curated from the Immunology Database and Analysis Portal (ImmPort; https://www.immport.org/home) (Bhattacharya et al., 2018). Through limma package (Ritchie et al., 2015), differentially expressed immune-associated genes were screened with 45 early-stage and 48 advanced-stage carotid atherosclerotic plaques in line with the criteria of |fold-change|>1.5 and false discovery rate (FDR) < 0.05. These genes were regarded as atherosclerotic plaque progression- and immune-associated genes.

### Functional Enrichment Analysis

ClusterProfiler package (Yu et al., 2012) was utilized for functionally analyzing the biological functions, which comprises Gene Ontology (GO) and Kyoto Encyclopedia of Genes and Genomes (KEGG). The *p*-value was adjusted using the Benjamini–Hochberg approach or FDR for multiple testing corrections. The threshold was set at FDR<0.05. GO categories comprised biological processes (BP), molecular functions (MF), and cellular components (CC).

### Protein–Protein Interaction (PPI)

The atherosclerotic plaque progression- and immune-associated genes were uploaded onto the online “Search Tool for the Retrieval of Interacting Genes” (STRING; http://string-db.org) and their interaction pairs were required. Through the plug-in of Cytoscape Molecular Complex Detection (MCODE) (Bader and Hogue, 2003), hub modules of the PPI network were established following the threshold of degree cutoff = 2, K-Core = 2, and node score cutoff = 0.2.

### Selection of Characteristic Genes

Three machine learning algorithms, LASSO, random forest, and SVM-RFE (Sanz et al., 2018), were applied for screening characteristic genes. LASSO, a dimension reduction approach, shows superiority in evaluating high-dimensional data in comparison to regression analysis. LASSO analysis was implemented with a turning/penalty parameter utilizing a 10-fold cross-verification via glmnet package (Engebretsen and Bohlin, 2019). Recursive feature elimination (RFE) from the random forest algorithm, an approach of supervised machine learning, was applied for ranking the atherosclerotic plaque progression- and immune-associated genes. The predictive performance was estimated via ten-fold cross-validation, and the genes with relative importance>0.25 were determined as the characteristic genes. SVM-RFE is superior to linear discriminant analysis and to the mean squared error method to select relevant characteristics and remove redundant characteristics. SVM-RFE was applied for feature selection via ten-fold cross-validation. Receiver operating characteristic (ROC) curves and the area under the curve (AUC) were used for estimating the diagnostic efficacy.

### Landscape of Immune Cell Infiltrations

Single-sample gene set enrichment analysis (ssGSEA) was implemented to analyze the infiltration levels of immune cells on the basis of the expression profiling of 29 immunity-relevant signatures.

### Gene Set Enrichment Analysis (GSEA)

GSEA was implemented for functionally elucidating the biological significance of characteristic genes (Subramanian et al., 2005). The gene set of “c2.cp.kegg.v11.0.symbols” from the Molecular Signature Database (MSigDB, http://software.broadinstitute.org/gsea/msigdb) (Liberzon et al., 2015) was utilized as the reference set. For achieving a normalized enrichment score for each analysis, gene set permutations with 1,000 times were conducted. An FDR<0.05 was regarded as significant enrichment.

### Establishment of a Nomogram

Characteristic genes were incorporated to establish a nomogram using the rms package. The calibration curve was utilized for evaluating the accuracy of the nomogram. Through the decision curve analysis, the clinical usefulness of the nomogram was evaluated.

### Prediction of Candidate Small-Molecule Compounds

The Connectivity Map (CMap, https://clue.io/), a web-based database, applies cellular responses to perturbations for finding interactions between diseases, genes, and small-molecule compounds (Subramanian et al., 2017). The atherosclerotic plaque progression- and immune-associated genes were interrogated to compare the similarity to all perturbed signatures in this database. The candidate small-molecule compounds were determined with an |enrichment score|>90. Moreover, compounds with positive or negative enrichment scores were selected for predicting the mode of action (MoA).

### Consensus Clustering Analysis

The consensus clustering approach was applied to quantitatively estimate the number of unsupervised classes across carotid atherosclerotic plaque specimens via the ConsensusClusterPlus package (50 iterations and resampling rate of 80%) on the basis of expression profiling of atherosclerotic plaque progression- and immune-associated genes (Wilkerson and Hayes, 2010). The consensus matrix plot, consensus cumulative distribution function (CDF) plot, relative alterations in area under the CDF curve, and tracking plot were implemented for finding the optimal number of clusters. Principal component analysis (PCA) was utilized for defining the expression difference in atherosclerotic plaque progression- and immune-associated genes between two subtypes. The PCA diagram was depicted utilizing the ggplot2 package (Ito and Murphy, 2013).

### Gene Set Variation Analysis (GSVA)

GSVA is a non-parametric and unsupervised gene set enrichment approach, which evaluates the association between biological pathways and gene signatures on the basis of expression profiling (Hänzelmann et al., 2013). Fifty hallmark gene sets were curated from the MSigDB as the reference set. The GSVA package and its ssGSEA function were applied for obtaining the GSVA score of each gene set. The GSVA score denoted the degree of absolute enrichment of each gene set. Limma package was utilized for comparing the difference in the GSVA score of each gene set between subtypes.

### Statistical Analysis

All statistical tests were implemented utilizing R software 3.6.1. Wilcoxon or Student’s t-test was utilized for analyzing the difference between the two groups. The correlation between the variables was determined using Pearson’s or Spearman’s correlation test. All statistical *p*-values were two-sided, and *p* < 0.05 was regarded as statistical significance.

## Results

### Identification of Atherosclerotic Plaque Progression- and Immune-Associated Genes

**FIGURE 1:**
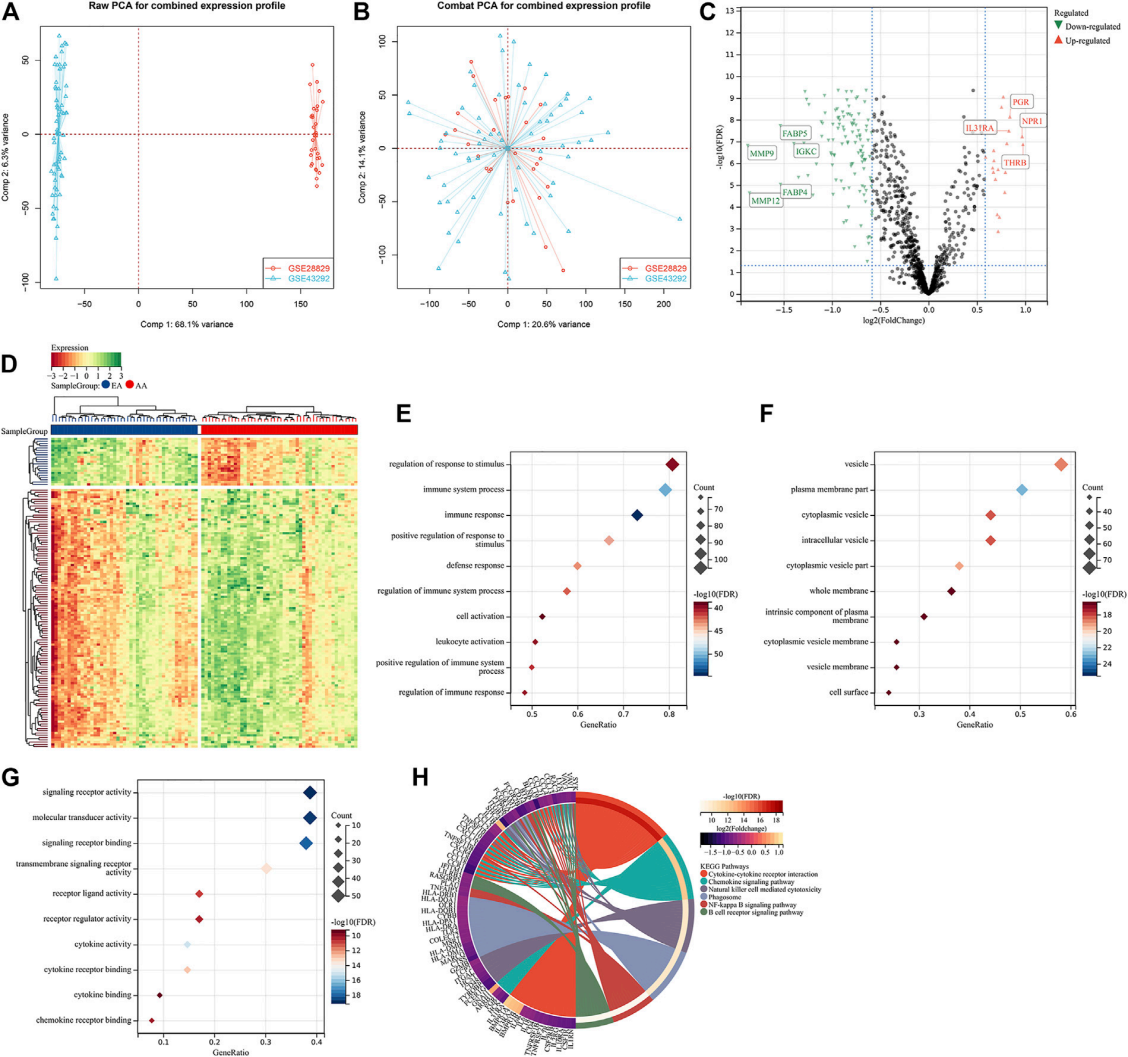
Identification of atherosclerotic plaque progression- and immune-associated genes in the combined expression profiling of GSE28829 and GSE43292 cohorts. **(A)** PCA plots showing the combined expression profiling of GSE28829 and GSE43292 cohorts. **(B)** PCA plots showing the combined expression profiling of GSE28829 and GSE43292 cohorts after batch effects. **(C)** Volcano plots depicting the RNA expression levels of the immune-associated genes between early early-stage and advanced-stage carotid atherosclerotic plaque specimens. **(D)** Heatmap showing the differentially expressed immune-associated genes between the aforementioned groups. AA: advanced-stage atherosclerotic plaque; EA: early-stage atherosclerotic plaques. **(E–G)** Main BPs, CCs, and MFs enriched by atherosclerotic plaque progression- and immune-associated genes. **(H)** Main KEGG pathways enriched by the above genes.

To investigate the roles of immune-associated genes in the progression of atherosclerotic plaques, we combined the expression profiles of 45 early-stage and 48 advanced-stage atherosclerotic plaque specimens from the GSE28829 and GSE43292 cohorts (Figure 1A). Batch effects were adjusted for subsequent analysis (Figure 1B). Among 1,242 immune-associated genes, 114 presented downregulation and 21 presented upregulation in an advanced-stage compared to early-stage atherosclerotic plaques (Figures 1C,D). The detailed information is listed in Supplementary Table 1. These atherosclerotic plaque progression- and immune-associated genes were linked to immune responses such as cytokine–cytokine receptor interaction and chemokine signaling pathway (Figures 1E–H).

### Identification of Hub Atherosclerotic Plaque Progression- and Immune-Associated Genes

**FIGURE 2:**
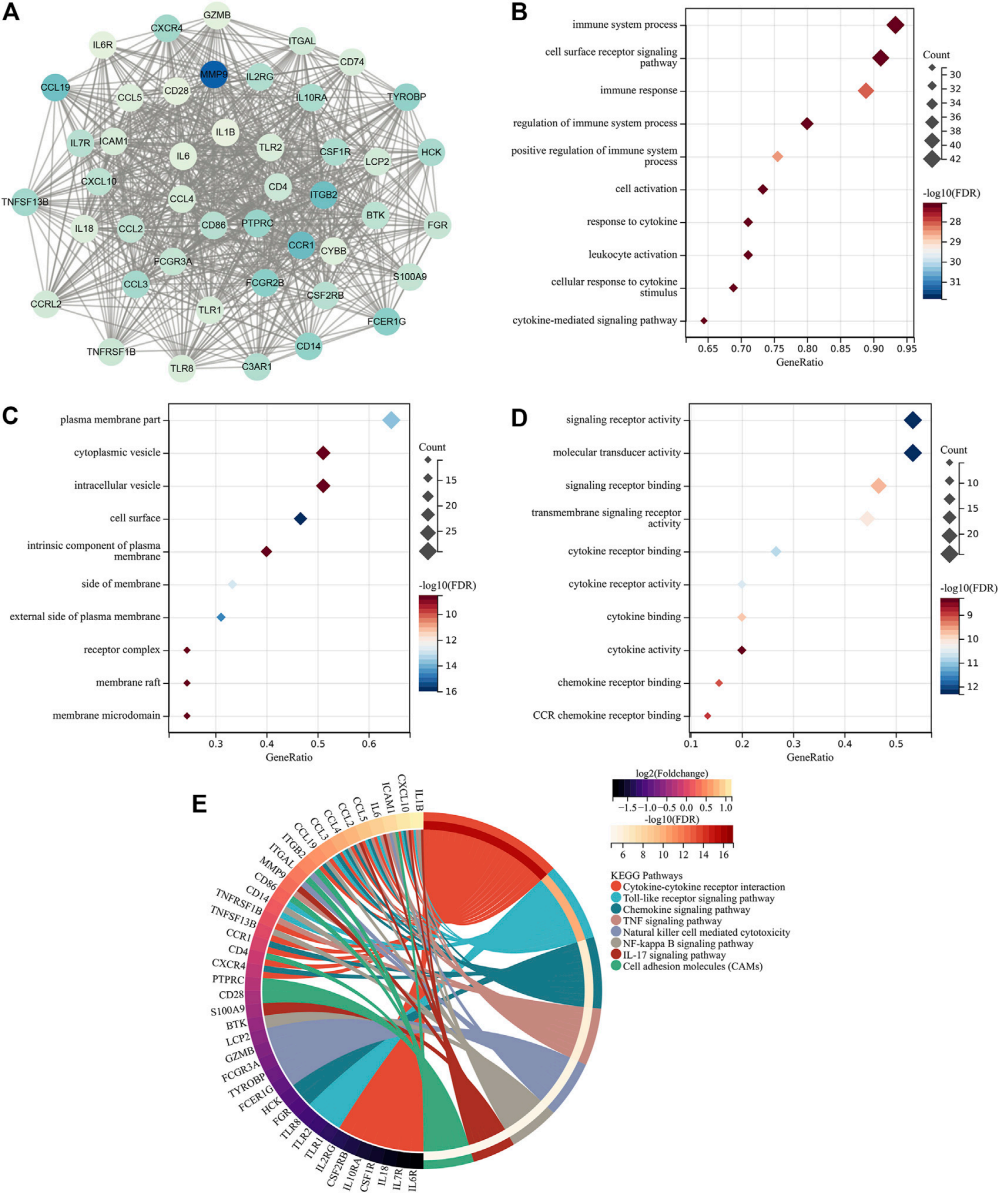
Identification of key atherosclerotic plaque progression- and immune-associated genes. **(A)** MCODE analysis identifies the hub module from the PPI network of atherosclerotic plaque progression- and immune-associated genes. **(B–D)** Main BPs, CCs, and MFs enriched by key atherosclerotic plaque progression- and immune-associated genes. **(E)** Main KEGG pathways enriched by the aforementioned genes.

Through MCODE analysis, one hub module from the PPI network was established, which comprised key atherosclerotic plaque progression- and immune-associated genes (Figure 2A). Further analysis displayed that they mainly participated in cytokine–cytokine receptor interaction, Toll-like receptor signaling pathway, chemokine signaling pathway, TNF signaling pathway, natural killer cell-mediated cytotoxicity, NF-kappa B signaling pathway, IL-17 signaling pathway, and cell adhesion molecules (Figures 2B–E), indicating the crucial roles of key atherosclerotic plaque progression- and immune-associated genes.

### Selection of Characteristic Genes via LASSO, Random Forest and SVM-RFE Algorithms

**FIGURE 3:**
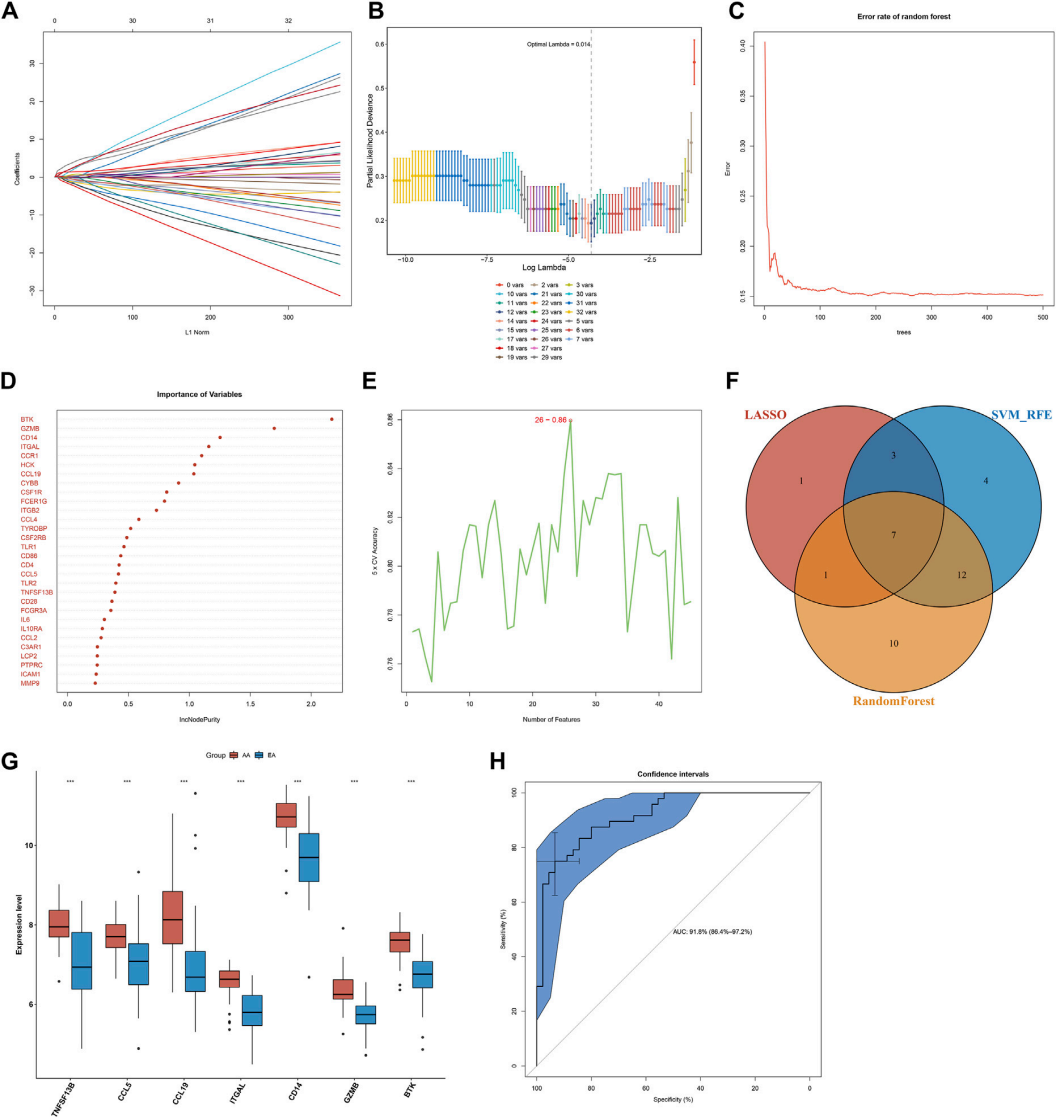
Selection of characteristic genes among key atherosclerotic plaque progression- and immune-associated genes and estimation of their diagnostic efficacy in the combined GSE28829 and GSE43292 datasets. **(A)** Ten-time cross-verification for tuning parameter selection in the LASSO model. Each curve corresponds to a single gene. **(B)** LASSO coefficient profiling. The solid vertical lines represent the partial likelihood deviance SE. The dotted vertical line is drawn at the optimal lambda. **(C)** Random forest for the relationships between the number of trees and error rate. **(D)** The rank of genes in accordance with their relative importance. **(E)** SVM-RFE algorithm for feature selection. **(F)** Venn diagram showing the characteristic genes shared by LASSO, random forest, and SVM-RFE algorithms. **(G)** Box plots depicting the mRNA expression of characteristic genes in early-stage and advanced-stage atherosclerotic plaques. AA: advanced-stage atherosclerotic plaque; EA: early-stage atherosclerotic plaques. ***p < 0.001. **(H)** The ROC curves estimating the diagnostic performance of characteristic genes.

Three algorithms were applied for selecting characteristic genes among key atherosclerotic plaque progression- and immune-associated genes. For the LASSO algorithm, the optimal lambda was 0.014 following ten-fold cross-validation. Thus, we chose the minimum criteria for building the LASSO classifier due to higher accuracy by comparisons, and 12 characteristic genes were identified, containing ITGAL, IL7R, IL18, CCL19, BTK, TLR8, CD14, CCL5, IL1B, IL6, GZMB, and TNFSF13B (Figures 3A,B). For the random forest algorithm, 30 characteristic genes with relative importance >0.25 were determined, including MMP9, ICAM1, PTPRC, LCP2, C3AR1, CCL2, IL10RA, IL6, FCGR3A, CD28, TNFSF13B, TLR2, CCL5, CD4, CD86, TLR1, CSF2RB, TYROBP, CCL4, ITGB2, FCER1G, CSF1R, CYBB, CCL19, HCK, CCR1, ITGAL, CD14, GZMB, and BTK (Figures 3C,D). For the SVM-RFE algorithm, when the feature number was 26, the classifier had the minimum error, containing CD14, ITGAL, TNFSF13B, IL18, CCL5, PTPRC, CCRL2, IL7R, MMP9, BTK, IL10RA, CD28, GZMB, ICAM1, HCK, CSF2RB, CD74, TLR2, CCR1, C3AR1, CCL19, IL2RG, TYROBP, CSF1R, CXCR4, and IL1B (Figure 3E). Following intersection, 7 characteristic genes shared by LASSO, random forest, and SVM-RFE algorithms were finally identified (TNFSF13B, CCL5, CCL19, ITGAL, CD14, GZMB, and BTK; Figure 3F).

### Diagnostic Efficacy of Characteristic Genes in Predicting Atherosclerotic Plaque Progression

**FIGURE 4:**
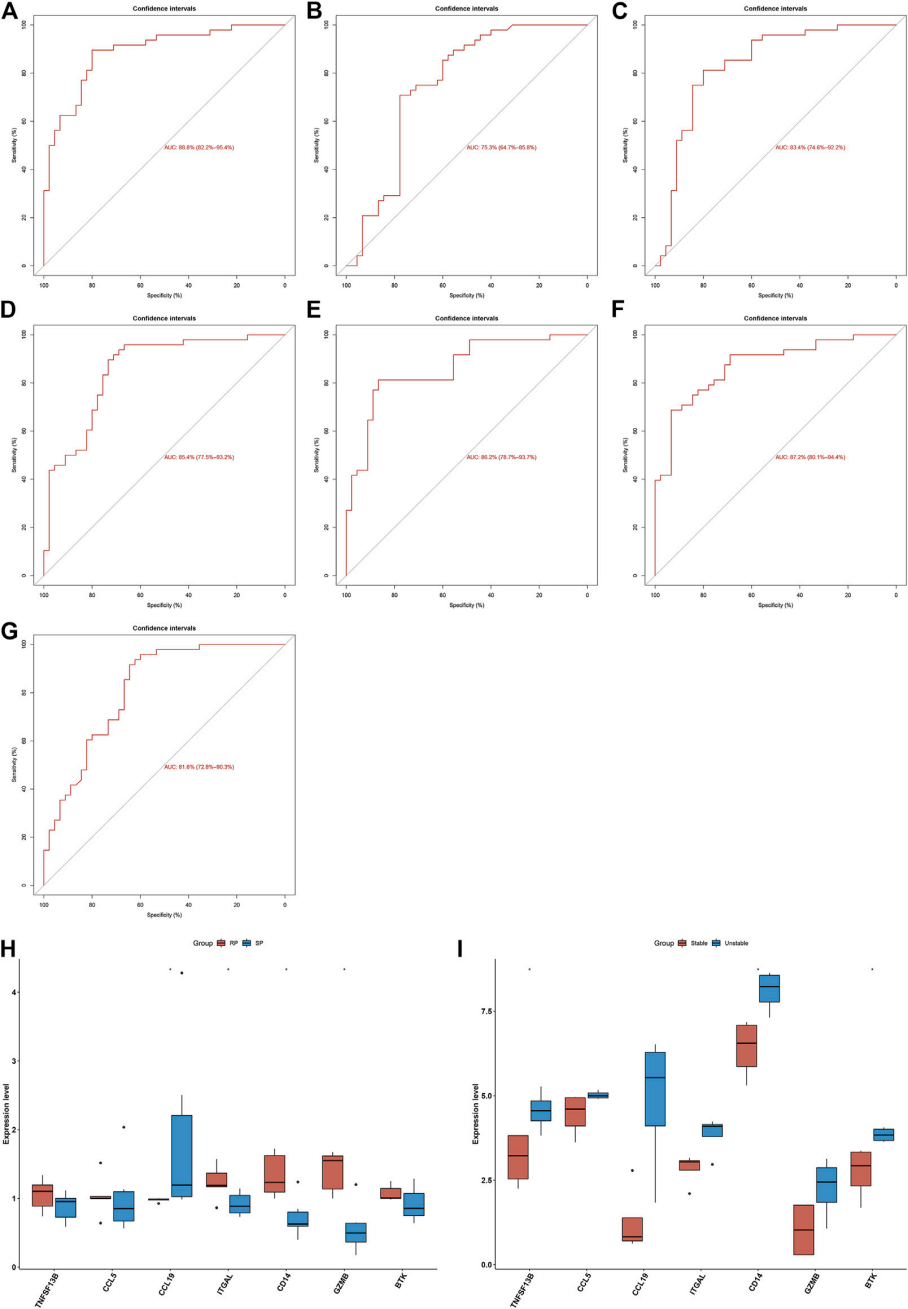
Diagnostic efficacy of characteristic genes in the prediction of atherosclerotic plaque progression and external verification of expression of characteristic genes. **(A–G)** ROC curves estimating the diagnostic performance of characteristic genes **(A)** BTK, **(B)** CCL5, **(C)** CCL19, **(D)** CD14, **(E)** GZMB, **(F)** ITGAL, and **(G)** TNFSF13B in predicting atherosclerotic plaque progression in the combined GSE28829 and GSE43292 datasets. **(H)** Box plots showing the mRNA expression of characteristic genes in ruptured and stable atherosclerotic plaque in the GSE41571 dataset. RP: ruptured plaque; SP: stable plaque. **(I)** Box plots showing the mRNA expression of characteristic genes in stable and unstable atherosclerotic plaque specimens in the GSE120521 dataset. *p < 0.05.

Seven characteristic genes (TNFSF13B, CCL5, CCL19, ITGAL, CD14, GZMB, and BTK) presented higher expression in advanced-stage than in early-stage atherosclerotic plaques (Figure 3G), indicating their potential roles during the progression of atherosclerotic plaques. When all of them were fitted into one variable, the AUC of the ROC curve was 0.918, demonstrating the favorable diagnostic efficiency in predicting atherosclerotic plaque progression (Figure 3H). We also estimated the diagnostic performance of each characteristic gene in predicting atherosclerotic plaque progression in the combined GSE28829 and GSE43292 cohorts. The AUC values of ROC curves were 0.888 of BTK (Figure 4A), 0.753 of CCL5 (Figure 4B), 0.834 of CCL19 (Figure 4C), 0.854 of CD14 (Figure 4D), 0.862 of GZMB (Figure 4E), 0.872 of ITGAL (Figure 4F), and 0.816 of TNFSF13B (Figure 4G), demonstrating that these characteristic genes enabled to estimate the progression of atherosclerotic plaques.

### External Validation of Diagnostic Performance of Characteristic Genes in Estimating Atherosclerotic Plaque Progression

The expression of characteristic genes was verified in external datasets. In the GSE41571 dataset, CCL19 presented higher expression in stable than in ruptured plaques, while ITGAL, CD14, and GZMB had higher expression in ruptured than in stable plaques (Figure 4H). In the GSE120521 dataset, higher TNFSF13B, CD14, and BTK expression was confirmed in unstable than in stable plaques (Figure 4I). The AUC values of ROC curves were 0.717 of BTK (Supplementary Figure 1A), 0.567 of CCL5 (Supplementary Figure 1B), 0.917 of CCL19 (Supplementary Figure 1C), 0.900 of CD14 (Supplementary Figure 1D), 0.933 of GZMB (Supplementary Figure 1E), 0.900 of ITGAL (Supplementary Figure 1F), and 0.700 of TNFSF13B (Supplementary Figure 1G) in the GSE41571 dataset, indicating their potential in distinguishing ruptured from stable plaques. Moreover, the AUC values of the ROC curves were 1.000 of BTK (Supplementary Figure 2A), 0.812 of CCL5 (Supplementary Figure 2B), 0.938 of CCL19 (Supplementary Figure 2C), 1.000 of CD14 (Supplementary Figure 2D), 0.875 of GZMB (Supplementary Figure 2E), 0.812 of ITGAL (Supplementary Figure 2F), and 0.969 of TNFSF13B (Supplementary Figure 2G) in the GSE120521 dataset, demonstrating that they are capable of differentiating unstable from stable plaques. Hence, the characteristic genes possessed excellent diagnostic performance in predicting the progression of atherosclerotic plaques.

### Alterations in Immunological Features From Early-Stage to Advanced-Stage Atherosclerotic Plaques

**FIGURE 5:**
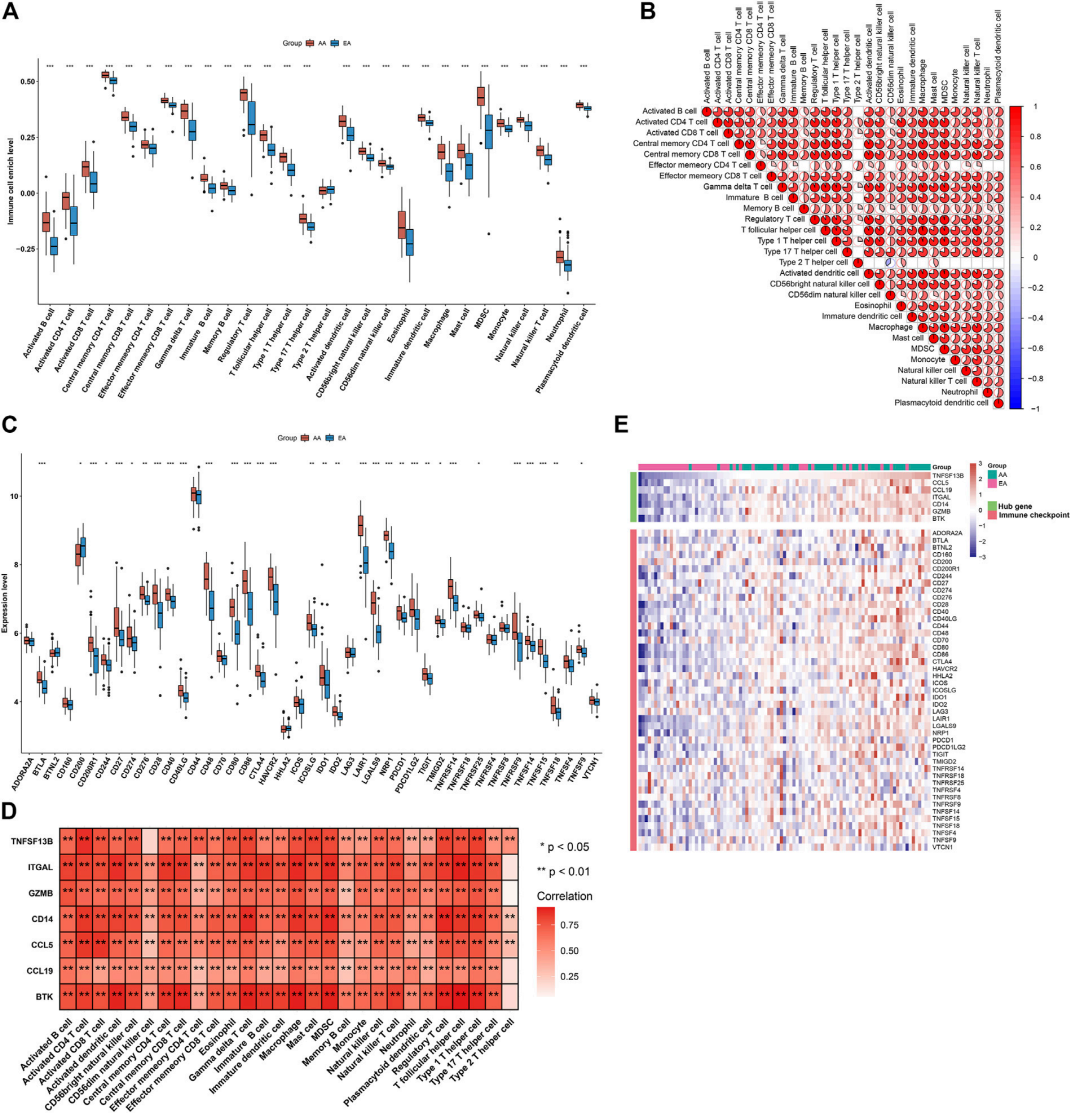
Alterations in the immunological features from early-stage to advanced-stage atherosclerotic plaques and correlations between the characteristic genes and immunological features in the combined GSE28829 and GSE43292 datasets. **(A)** Box plots depicting the infiltration levels of immune cells in early-stage and advanced-stage atherosclerotic plaques. **(B)** Heatmaps depicting the correlations between distinct immune cell compositions. **(C)** Box plots showing the mRNA expression of immune checkpoints in early-stage and advanced-stage atherosclerotic plaques. **(D)** Correlation analysis of immune cell infiltrations with characteristic genes. **(E)** Visualization of the relationships between immune checkpoints and characteristic genes. *p < 0.05; **p < 0.01; and ***p < 0.001.

Immunological features were evaluated in accordance with immune cell infiltration and immune checkpoint expression. Compared with early-stage atherosclerotic plaques, most innate and adaptive immune cells presented higher infiltration levels in advanced-stage atherosclerotic plaques (Figure 5A). Moreover, there were remarkable interactions between immune cell populations across atherosclerotic plaques (Figure 5B). As illustrated in Figure 5C, the higher expression of most immune checkpoints was investigated in advanced-stage than in early-stage atherosclerotic plaques. The aforementioned data indicated a higher immune response in advanced-stage atherosclerotic plaques. Furthermore, analyses displayed positive interactions between characteristic genes and immune cell infiltrations (Figure 5D). Additionally, characteristic genes were positively linked to immune checkpoints across atherosclerotic plaques (Figure 5E). Hence, the characteristic genes might modulate immunological features during atherosclerotic plaque progression.

### Signaling Pathways Involved in Characteristic Genes

**FIGURE 6:**
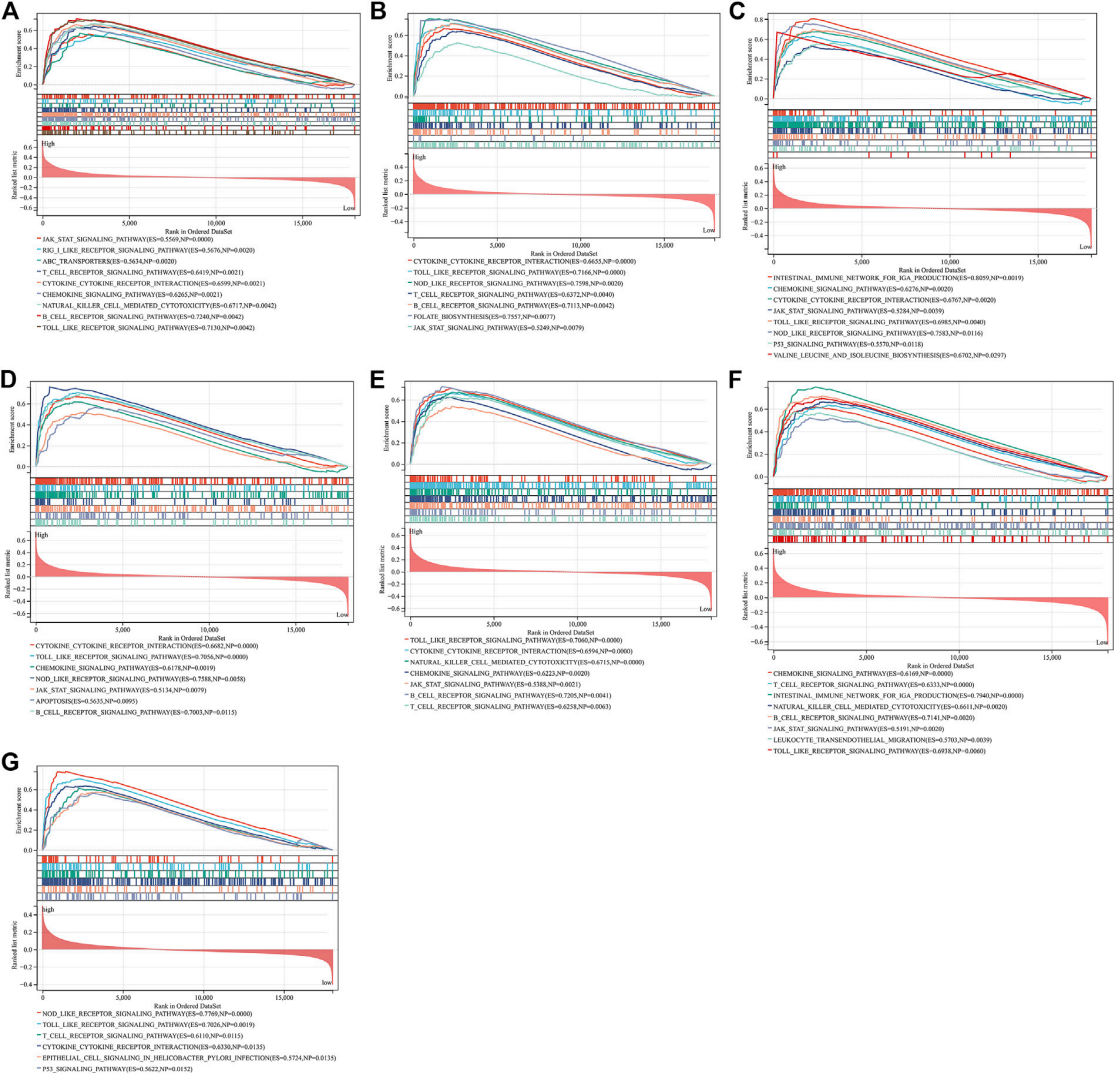
GSEA identifies signaling pathways involved in the characteristic genes. **(A–G)** The main signaling pathways that are significantly enriched in high expressions of characteristic genes **(A)** BTK, **(B)** CCL5, **(C)** CCL19, **(D)** CD14, **(E)** GZMB, **(F)** ITGAL, and **(G)** TNFSF13B.

Through GSEA, we evaluated signaling pathways involved in the characteristic genes. Our results demonstrated that BTK (Figure 6A), CCL5 (Figure 6B), CCL19 (Figure 6C), CD14 (Figure 6D), GZMB (Figure 6E), ITGAL (Figure 6F), and TNFSF13B (Figure 6G) were all positively linked to the immune responses (cytokine–cytokine receptor interaction, Toll-like receptor signaling pathway, B-cell or T-cell receptor signaling pathway, etc.).

### Establishment of a Characteristic Gene-Based Nomogram for Predicting Atherosclerotic Plaque Progression

**FIGURE 7:**
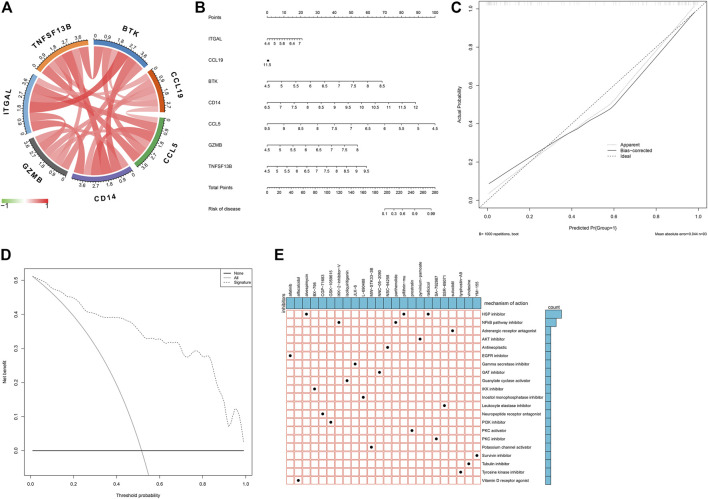
Establishment of a characteristic gene-based nomogram and selection of potential small molecular compounds. **(A)** Interactions between characteristic genes at the molecular level. **(B)** Establishment of a nomogram integrating characteristic genes for predicting atherosclerotic plaque progression. In the nomogram, each variable corresponds to a score, and the total score can be calculated by adding the scores for all variables. **(C)** Calibration curve estimates the prediction accuracy of the nomogram. **(D)** Decision curve analysis shows the clinical benefit of the nomogram. **(E)** The mechanisms of action shared by small molecular compounds based on CMap analysis.

As illustrated in Figure 7A, there were remarkable interactions between the characteristic genes. By incorporating characteristic genes, a nomogram was constructed as a diagnostic tool for atherosclerotic plaque progression (Figure 7B). In the nomogram, each characteristic gene corresponded to a score, and the total score was calculated by adding the scores for all characteristic genes. The total points corresponded to different risks of atherosclerosis. The calibration curve demonstrated that the nomogram enabled an accurate estimation of the progression of atherosclerotic plaques (Figure 7C). As depicted in the decision curve analysis, the patients diagnosed with atherosclerosis could clinically benefit from the nomogram (Figure 7D).

### Prediction of Small Molecular Compounds Against Atherosclerosis Based on Atherosclerotic Plaque Progression- and Immune-Associated Genes

On the basis of atherosclerotic plaque progression- and immune-associated genes, potential small molecular compounds against atherosclerosis were predicted through CMap analysis, as depicted in Figure 7E. Among them, alvespimycin, pifithrin-mu, and radicicol shared HSP inhibitors, while IKK-2-inhibitor-V and radicicol shared NF-kappa B pathway inhibitors.

### Construction of Two Immune Subtypes of Atherosclerosis Based on Atherosclerotic Plaque Progression- and Immune-Associated Genes

**FIGURE 8:**
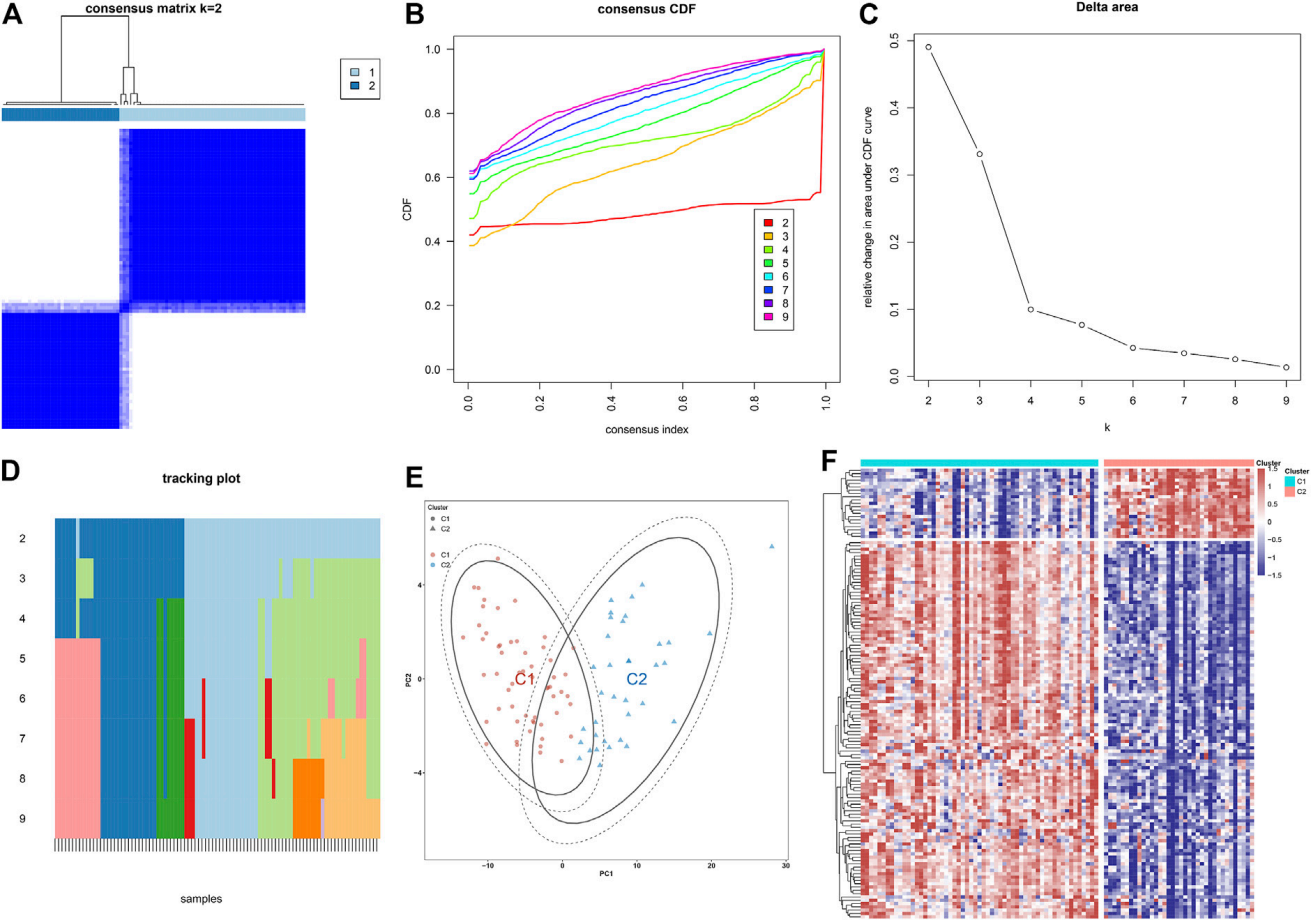
Construction of two immune subtypes of atherosclerosis based on atherosclerotic plaque progression- and immune-associated genes in the combined GSE28829 and GSE43292 datasets. **(A)** Consensus matrix heatmap when k = 2. **(B)** Consensus CDF when k = 2–9. **(C)** Relative alterations in the area under CDF curve. **(D)** Tracking plot showing the sample classification when k = 2–9. **(E)** PCA plots demonstrating that atherosclerotic plaque specimens are categorized as two immune subtypes (C1, C2) in accordance with the expression profiling of atherosclerotic plaque progression- and immune-associated genes. **(F)** Heatmap showing the expression of atherosclerotic plaque progression- and immune-associated genes in two immune subtypes.

Through the consensus clustering approach, atherosclerotic plaques were clustered in accordance with expression profiling of 135 atherosclerotic plaque progression- and immune-associated genes. The optimal number of subtypes was 2, which was determined using a consensus matrix plot, a CDF plot, relative alterations in the area under the CDF curve, and a tracking plot (Figures 8A–D). The two immune subtypes were named C1 and C2. PCA demonstrated the remarkable difference between the subtypes (Figure 8E). It was found that there was remarkable heterogeneity in the expression of atherosclerotic plaque progression- and immune-associated genes between subtypes (Figure 8F).

### Two Immune Subtypes Characterized by Different Immunological Features and Molecular Mechanisms

**FIGURE 9:**
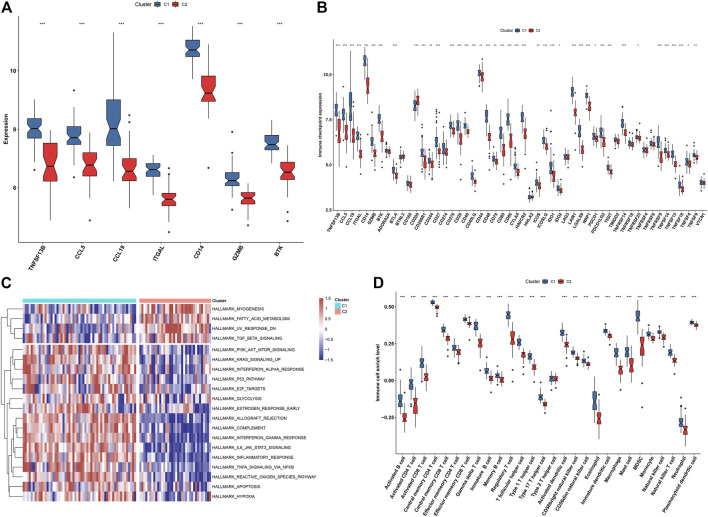
Two immune subtypes characterized by different immunological features and molecular mechanisms. **(A)** Box plots showing the mRNA expression of characteristic genes in two immune subtypes. **(B)** Box plots showing the mRNA expression of immune checkpoints in two immune subtypes. **(C)** Heatmap showing the enrichment levels of hallmark gene sets in two immune subtypes. **(D)** The box plots demonstrating the infiltration levels of immune cell components in two immune subtypes. *p < 0.05; **p < 0.01; and ***p < 0.001.

In Figure 9A, we noticed that all characteristic genes presented a higher expression in C1 than C2 subtype. In comparison to the C2 subtype, most immune checkpoints were remarkably upregulated in the C1 subtype (Figure 9B). As illustrated in Figure 9C, the C1 subtype had higher immune activation (allograft rejection, complement, interferon-gamma response, IL6-JAK-STAT3 signaling, inflammatory response, TNFα signaling via NF-kappa B, etc.) than the C2 subtype. Further analysis demonstrated that the C1 subtype presented higher infiltration levels of most immune cell populations than the C2 subtype (Figure 9D). Collectively, we identified C1 as an immune subtype and C2 as a non-immune subtype.

## Discussion

Experimental and clinical evidence has demonstrated that atherosclerosis represents a chronic inflammatory disease resulting in the formation of atherosclerotic plaques at specific sites. Hence, it is of importance to develop novel diagnostic tools for risk stratification of atherosclerotic plaques. Except for human cancers (Chen et al., 2020; Kong et al., 2020; Qiu et al., 2020), both innate and adaptive immune mechanisms enable the facilitation or control of atherosclerosis. It is significant to uncover the roles of immune-associated genes during the progression of atherosclerotic plaques.

In the combined expression profiling of 45 early-stage and 48 advanced-stage atherosclerotic plaques from the GSE28829 and GSE43292 datasets, we determined 114 downregulated and 21 upregulated immune-associated genes in advanced-stage compared to early-stage atherosclerotic plaques. On the basis of three machine learning algorithms, we selected seven characteristic genes (TNFSF13B, CCL5, CCL19, ITGAL, CD14, GZMB, and BTK). All of them enabled us to precisely predict the progression of atherosclerotic plaques. Limited evidence suggested the roles of the characteristic genes in atherosclerosis. The persistent accumulation of the macrophages within the arterial intima from the onset of the disease is one of the hallmarks of atherosclerosis. The recruitment of monocytes results in the enhanced infiltration of macrophages at an early-stage atherosclerosis, which can be mediated by myeloid cell-derived CCL5 (Jongstra-Bilen et al., 2021). CCL19 modulates the inflammatory milieu in atherosclerotic lesions (Akhavanpoor et al., 2014), and its upregulation exerts an underlying pathogenic role in plaque destabilization (Damås et al., 2007). Moreover, CCL19 is upregulated in carotid atherosclerosis, and it enables the enhancement of proliferative capacity and MMP-1 expression in vascular smooth muscle cells, thereby contributing to the pro-atherogenic potential (Halvorsen et al., 2014). CD14 is involved in mediating the formation of macrophage foam cells (An et al., 2017). The preclinical animal models have revealed the significance of GZMB in atherosclerosis (Zeglinski and Granville, 2020). Moreover, BTK triggers atherosclerotic plaque formation by mediating oxidative stress, mitochondrial damage, and endoplasmic reticulum stress of macrophages (Qiu et al., 2021).

The microenvironment of atherosclerotic plaques comprises distinct innate and adaptive immune cells (Wolf and Ley, 2019). Most innate and adaptive immune cells had higher infiltrations in advanced-stage than in early-stage atherosclerotic plaques. Immune checkpoint blockade (ICB) treats an expanding range of human cancers (Liu et al., 2020; Chen et al., 2021; Niu et al., 2022), and the same checkpoints are crucial negative regulatory factors of atherosclerosis. In the matched cohort, cardiovascular events had an increased risk following ICB, and ICB was linked with atherosclerotic plaque progression (Drobni et al., 2020). We noticed a higher expression of most immune checkpoints in advanced-stage than in early-stage atherosclerotic plaques. The characteristic genes were positively linked to immune cell infiltrations and immune checkpoints across atherosclerotic plaques, indicating their roles in modulating immune activation during atherosclerotic plaque progression. Moreover, several small-molecule compounds were screened in accordance with atherosclerotic plaque progression- and immune-associated genes, such as alvespimycin, pifithrin-mu, and radicicol. However, experiments are required for the preliminarily evaluation of the therapeutic effects of these compounds in alleviating atherosclerosis.

We constructed two subtypes in accordance with expression profiling of atherosclerotic plaque progression- and immune-associated genes. The C1 immune subtype presented higher immune cell infiltrations and increased immune checkpoint expression than the C2 non-immune subtype. Thus, our classification enabled us to reflect the immune landscape of atherosclerotic plaques, which might assist the early diagnosis and intervention of atherosclerosis treatment. Despite this, several limitations should be pointed out. Although we identified characteristic atherosclerotic plaque progression- and immune-associated genes on the basis of machine learning algorithms and verified their diagnostic efficacy in external datasets, prospective cohorts will be conducted to further investigate the potential of the characteristic genes in predicting the progression of atherosclerotic plaques. Moreover, experiments will be presented to further clarify the mechanisms underlying the characteristic genes.

## Conclusion

Our findings determined seven characteristic atherosclerotic plaque progression- and immune-associated genes (TNFSF13B, CCL5, CCL19, ITGAL, CD14, GZMB, and BTK) that could predict the progression of atherosclerotic plaques. Moreover, we proposed a new molecular classification comprising immune and non-immune subtypes across atherosclerotic plaques. Collectively, our findings might assist in designing more precisely tailored cardiovascular immunotherapy.

## Data Availability

The datasets presented in this study can be found in online repositories. The names of the repository/repositories and accession number(s) can be found in the article/Supplementary Material.

## Abbreviations

LASSO: least absolute shrinkage and selection operator; SVM-RFE: support vector machine–recursive feature elimination; GEO: Gene Expression Omnibus; PCA: principal component analysis; ImmPort: Immunology Database and Analysis Portal; FDR: false discovery rate; GO: Gene Ontology; KEGG: Kyoto Encyclopedia of Genes and Genomes; BP: biological process; MF: molecular function; CC: cellular component; PPI: protein–protein interaction; STRING: Search Tool for the Retrieval of Interacting Genes; MCODE: molecular complex detection; RFE: recursive feature elimination; ROC: receiver operating characteristic; AUC: area under the curve; ssGSEA: single-sample gene set enrichment analysis; GSEA: gene set enrichment analysis; MSigDB: Molecular Signature Database; CMap: Connectivity Map; MoA: mode of action; CDF: cumulative distribution function; GSVA: gene set variation analysis.
