# Supplementary figures and images for: Immune-Associated Gene Signatures and Subtypes to Predict the Progression of Atherosclerotic Plaques Based on Machine Learning
Source: Front Pharmacol. 2022 Apr 26;13:865624. doi: 10.3389/fphar.2022.865624 (PMC9086243; doi:10.3389/fphar.2022.865624)

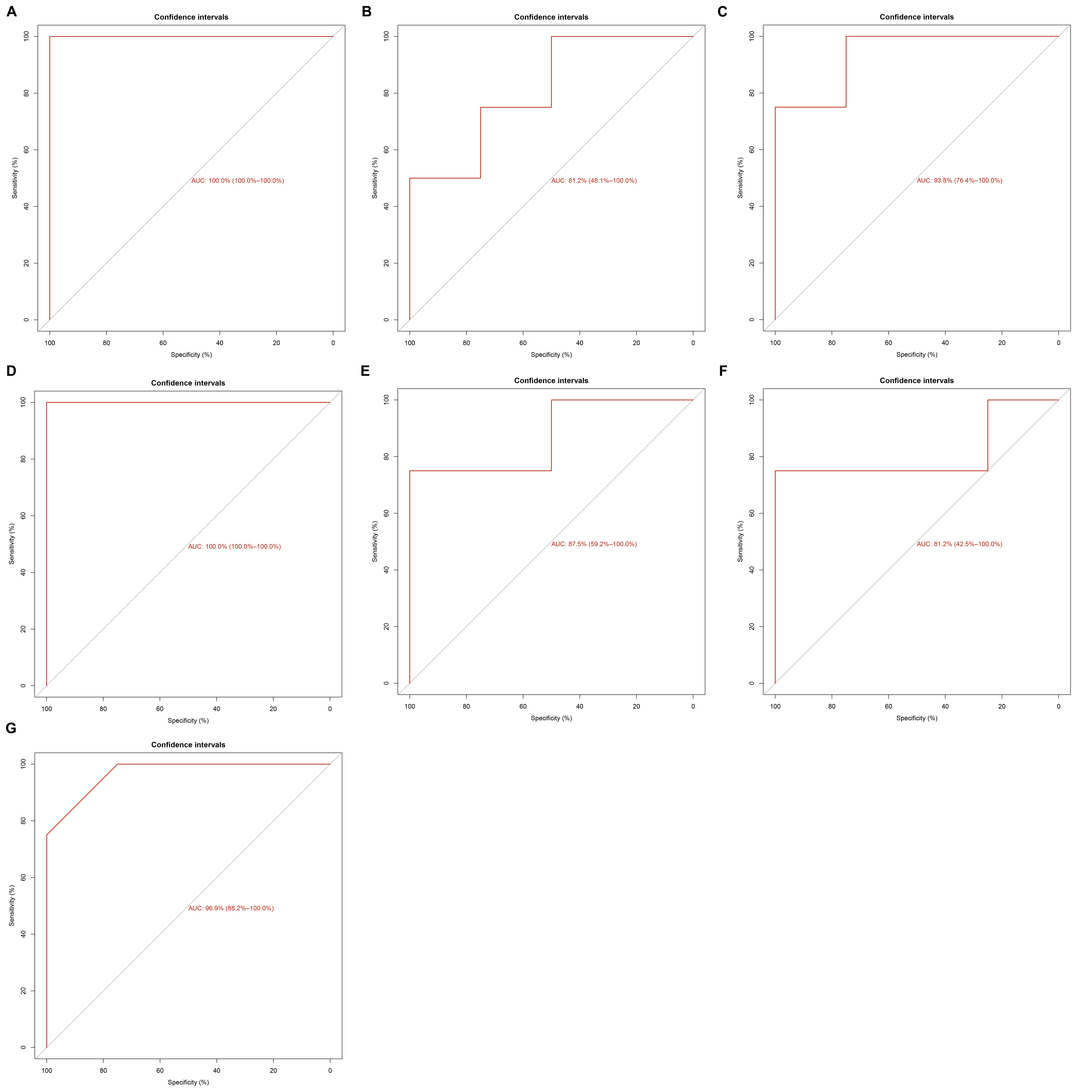

Supplement: Supplementary file 1 [file Image2.TIF]

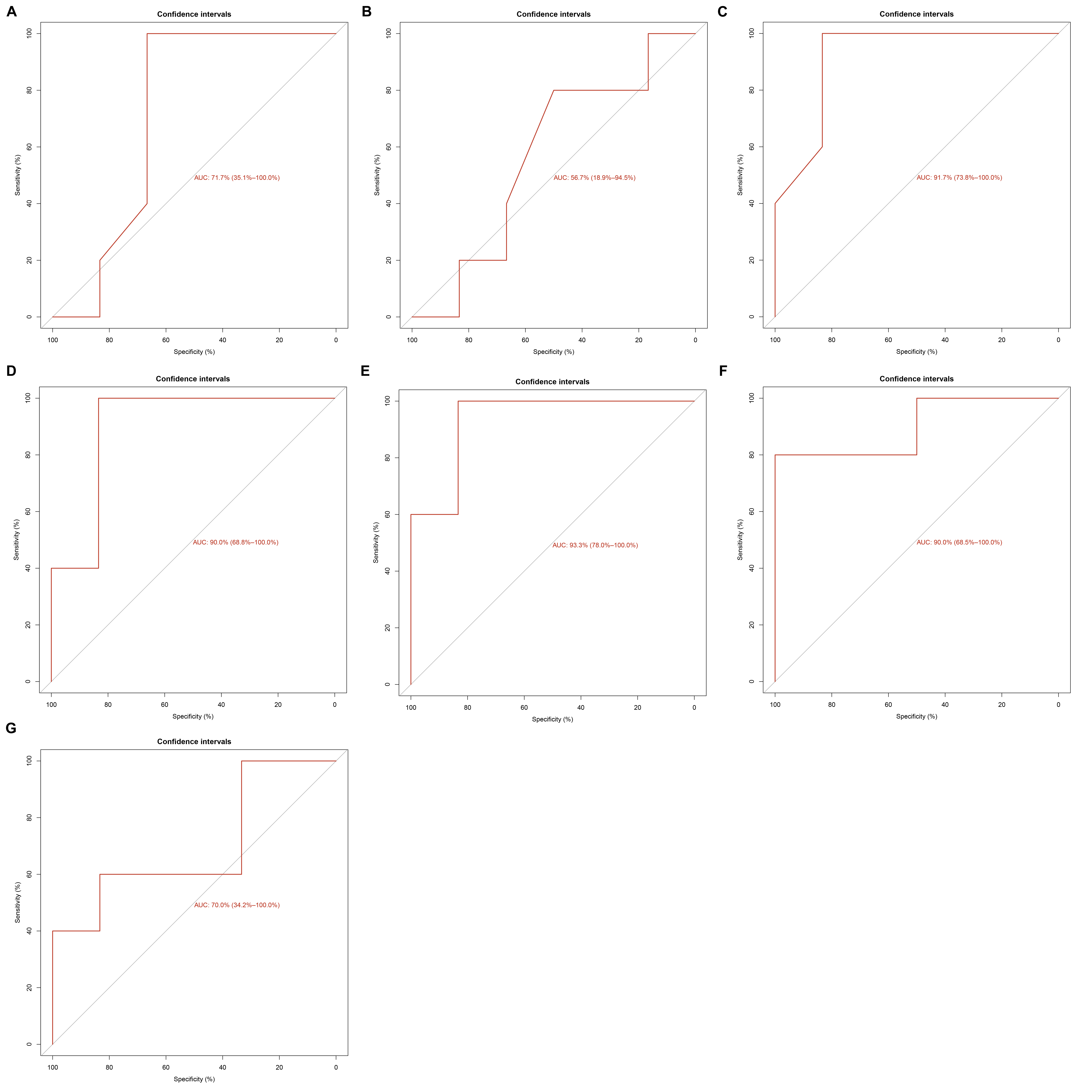

Supplement: Supplementary file 2 [file Image1.TIF]
